# Anterior Esthetic Restorations with the Stratified Stamp Technique: A Case Report

**DOI:** 10.3390/biomimetics9050299

**Published:** 2024-05-18

**Authors:** Camillo D’Arcangelo, Matteo Buonvivere, Francesco De Angelis

**Affiliations:** Unit of Restorative Dentistry and Endodontics, Department of Medical, Oral and Biotechnological Science, School of Dentistry, “G. D’Annunzio” University Chieti-Pescara, 66100 Chieti, Italy; cdarcang@unich.it (C.D.); matteo.buonvivere@unich.it (M.B.)

**Keywords:** dental trauma, direct composite veneer, case report

## Abstract

Anterior teeth restoration represents a challenge for dentists, who often rely on the dental technician’s wax-up. The proposed Stratified Stamp Technique (SST) allows for clinically reproducing the wax-up in a quick and easy way. A patient with fractures and discoloration on the upper central incisors was treated with resin-based composite direct restorations. Using SST, a 1 mm thick thermoformed polyethylene-terephthalate-glycol (PETG) template, based on the technician’s wax-up, was produced. Enamel Selective Area Reduction (SAR) was performed to guarantee adequate space for the restorations, and the fracture margins were rounded and finished. Traditional layering procedures according to the five color dimensions of teeth were performed, except for the final labial layer, which was realized with warm composite loaded inside the template and polymerized through it, in order to ensure accurate tooth morphology reproduction. SST offers a reliable method for transferring technician’s wax-up morphology to direct composite restorations in anterior teeth. Compared with other methods, SST allows for better isolation with a rubber dam and permits traditional layering with multiple composite shades, thus leading to satisfactory esthetic outcomes.

## 1. Introduction

Despite the progress in the mechanical performance and biocompatibility of dental materials, as well as the development of simplified techniques [1,2,3,4], anterior teeth restoration still represents one of the most difficult challenges for a general dentist.

Modern adhesive dentistry allows for esthetically restoring even severely compromised anterior teeth through minimally invasive direct approaches with resin-based composites [5]. In many clinical cases, this represent a valid and more conservative option to adhesively luted lithium disilicate or high translucent zirconia indirect restorations [6,7].

When restoring anterior teeth, the esthetic result depends on several factors: the appropriate shade selection, the layering technique, and a proper reproduction of tooth macro- and micro-morphology (superficial texture). While composite color matching and layering are nowadays eased by technology and handier materials [1,2], morphology reproduction still relies on clinician experience and the ability to follow the guide of the dental technician’s wax-up.

Several methods have been developed to transfer the tooth morphology directly from the wax-up to the direct restoration, such as the “injection molding technique” [8,9], relying on the injection of a flowable composite into a transparent silicone index based on the wax-up, and the “index technique”, based on the application of a warm conventional composite into a transparent silicone index separated for each tooth [10]. However, despite being quick and relatively easy, the above-mentioned techniques have several disadvantages, such as the need to use a single shade of composite without layering. The new “Stratified Stamp Technique” (SST) can overcome these limitations by merging conventional composite stratification with warm composite stamping. Using SST, in fact, direct restoration of two fractured incisors is performed by means of traditional composite layering with a silicone palatal key, except for the final labial layer, which is realized with a warm composite loaded inside a thermoformed polyethylene-terephthalate-glycol (PETG) template and polymerized through it. In this way, accurate tooth morphology reproduction is guaranteed without jeopardizing the natural chromaticity, value, intensives, opalescence, and characterizations [11] carefully replicated through composite layering.

## 2. Case Report

A 32-year-old man was referred to the dental clinic with the chief complaint of being unsatisfied with the esthetic of his smile. The patient’s medical history was unremarkable and he was taking no medication. The patient reported a history of a dental trauma due to a fall.

**Figure 1:**
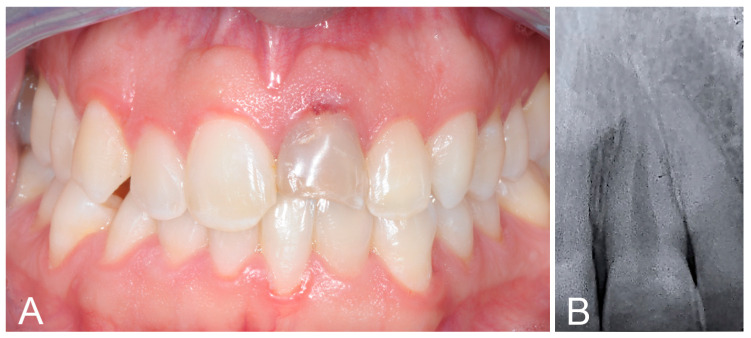
(**A**) Intraoral photo showing an enamel fracture on tooth 1.1 and an enamel–dentin fracture with severe discoloration on tooth 2.1. (**B**) Radiograph showing radiolucency on tooth 2.1.

The intraoral examination revealed fractures on both the upper central incisors: tooth 1.1 showed enamel fracture, while tooth 2.1 displayed enamel–dentin fracture and severe discoloration (Figure 1A). Teeth vitality was assessed using electric pulp testing (Digitest 3 Pulp Vitality Tester, Parcel Inc., Brentwood, NY, USA), which revealed a positive response for 1.1 and a negative one for 2.1. A radiograph displayed the presence of a periapical radiolucency on 2.1 (Figure 1B) and a diagnosis of asymptomatic apical periodontitis was formed.

**Figure 2:**
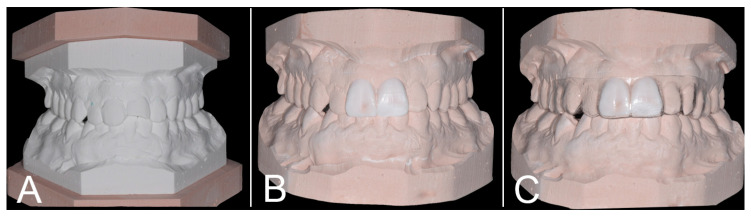
(**A**) Plaster study model of the patient’s upper and lower arch. (**B**) Additive wax-up of the central incisors made on a plster study model duplicate. (**C**) 1 mm thick thermoformed polyethylene-terephthalate-glycol template copying the shapes of the wax-up.

The proposed treatment plan consisted of root canal treatment for 2.1, followed by internal bleaching with 35% hydrogen peroxide solution, and direct resin-based restorations on both 1.1 and 2.1. Direct composite veneers on the anterior sextant were proposed as well, but the patient agreed to treat just the central incisors. After the root canal treatment, an impression was taken using polyvinylsiloxane material [12] (Aquasil Ultra+, Dentsply Sirona, Charlotte, NC, USA) in order to obtain a plaster study model to be used for esthetic and functional evaluations. An additive wax-up was then realized on a duplicated plaster model (Figure 2A,B). Specifically, the upper model was placed in a lab surveyor to mark the line of maximum convexity of the central incisors. This line, which is the consistently irregular border between the buccal and cervical sides of the frontal teeth, was used in the wax-up as a natural tooth-restoration transition margin, in order to avoid any over contouring following a concept already described for indirect anterior restorations [13,14]. Using a 1 mm thick PETG disc (Erkodur, Erkodent Erich Kopp GmbH, Pfalzgrafenweiler, Germany), a thermoformed template copying the shapes of the wax-up was then produced, to be used in the restorative phase (Figure 2C).

**Figure 3:**
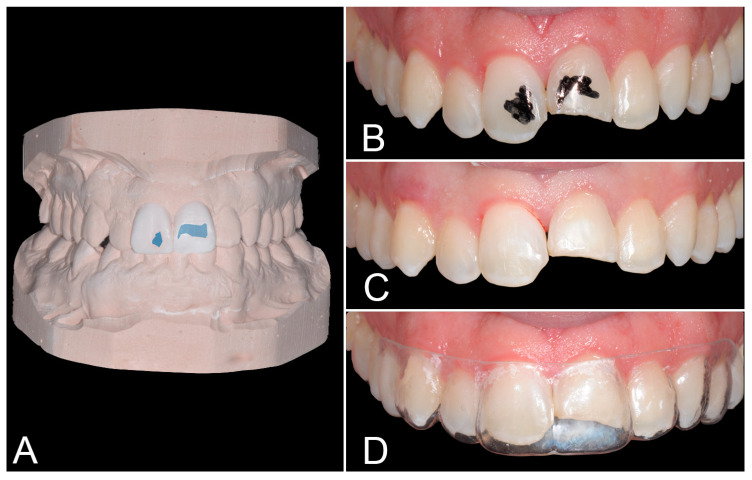
Areas of enamel to be reduced marked on both (**A**) the wax-up and (**B**) the patient’s teeth. (**C**) Enamel Selective Area Reduction performed and fracture margins rounded and finished. (**D**) Passive fit of the template confirmed.

Based on a completely additive wax-up, the template could already be inserted in the patient’s mouth without any preparation. However, in order to guarantee the minimal composite thickness all over the buccal face without a bulky appearance, some areas of enamel needed to be reduced. These areas were marked on both 1.1 and 2.1 (Figure 3A,B) and were subjected to a careful progressive reduction through fine grit bur until a perfect template fit was achieved, thus securing enough space for the composite material in each of the areas of teeth that needed to be restored. This procedure, named Selective Area Reduction (SAR), allowed for cutting as little enamel as possible so as to guarantee adequate space for the restorative material and to transfer the wax-up in the patient’s mouth in a minimally invasive way. The fractured enamel on the incisal edges was rounded and finished as well (Figure 3C), and the passive fit of the template was confirmed (Figure 3D).

Subsequently, under proper rubber dam isolation, 1.1 and 2.1 were sandblasted and etched, and adhesive procedures were carried out. Subsequently, resin-based composite layering procedures according to the five color dimensions of teeth [11] were performed. After the creation of the palatal composite shell (Enamel Plus Hri Function, shade UE2; Micerium S.p.A., Avegno, Italy) with the aid of a silicon index made from the same wax-up used for the template, two layers of dentinal core (Enamel Plus Hri Function, shades BD5 and BD4) were stratified in sequence in order to progressively reduce the chroma towards the incisal margin. Care was taken to sculpt the mamelons, leaving enough space in the incisal area to place a small amount of translucent material (Enamel Plus Hri Function, shade OBN), thus reproducing the natural blue opalescence of the incisal margin. At this point, no freehand stratification of the final buccal layer was performed. Instead, prior to the labial finalization of tooth 2.1, teeth 1.1 and 2.2 were protected with Teflon tape. A small amount of enamel composite (shade UE2) was warmed up to 55° C and then used to load the PETG template on tooth 2.1. Then, the PETG template was slowly inserted and pressure was progressively applied to ensure proper seating and to allow for the complete outflow of any excess material prior to prolonged polymerization. Upon template removal, the labial anatomy was perfectly defined, with an already polished and shiny surface.

Any interproximal or cervical flash material was carefully removed without employing burs or rotary tools, but with the simple use of hand instruments: a n. 15C surgical blade, metal (HORICO, Hopf, Ringleb & Co., GmbH & CIE, Berlin, Germany) and polyester (Sof-Lex, 3M, Saint Paul, MN, USA) interproximal strips. The same procedure was then repeated for tooth 1.1, while protecting teeth 1.2 and 2.1 with Teflon.

**Figure 4:**
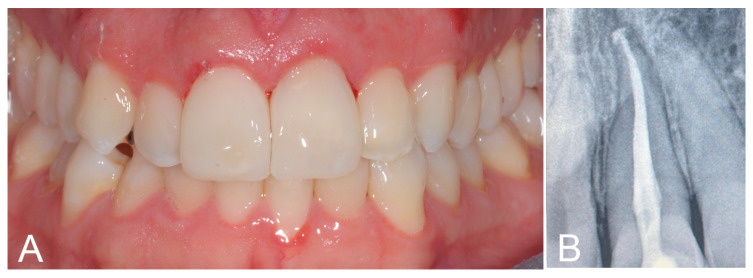
(**A**) Final esthetic result. (**B**) Radiographic check for the absence of any excess material in the cervical area.

Upon the rubber dam removal, further interproximal and cervical finishing was performed, occlusion was checked, and polishing procedures were performed just on the palatal side. The labial surfaces were left untouched (Figure 4A), thanks to their already polished and glossy appearance. The whole step-by-step procedure is showed in Video S1 in the Supplementary Materials. A final periapical radiograph was taken to exclude the presence of any excess material (Figure 4B).

**Figure 5:**
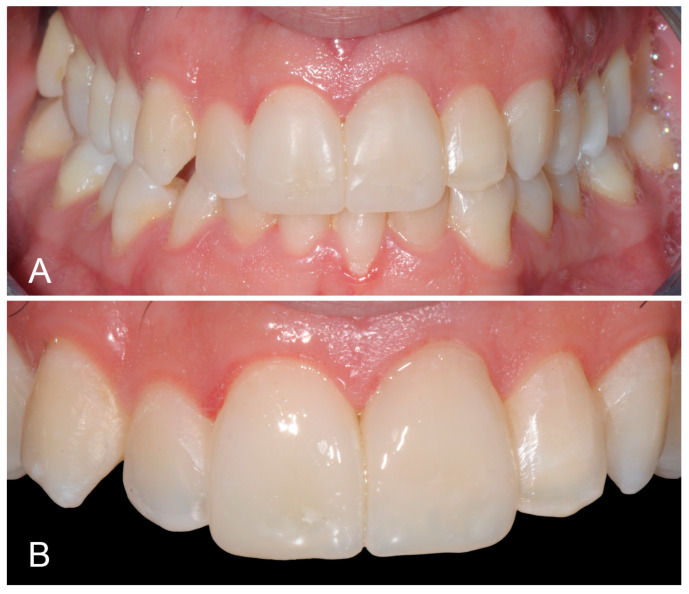
(**A**) One-year clinical follow-up showing a well preserved and stable esthetic outcome. (**B**) Upper front teeth at the 1-year follow-up.

At the 1-year clinical follow-up, the achieved esthetic outcome appeared well preserved and stable (Figure 5).

## 3. Discussion

Despite material and technique advancements, anterior restorations are still among the most challenging clinical procedures. Nowadays, color shade selection is eased by the use of custom shade guides [15], or even technologically aided by digital photocolorimetric/spectrophotometric analyses [16]. Shade matching alone is not enough to obtain esthetically pleasant restorations. The perceived esthetic of a tooth is, in fact, the result of the interaction of light with natural enamel and dentin, determining the so-called five color dimensions [11]. Accurate composite layering based on pre-operative pictures is, therefore, crucial to reproduce natural chromaticity, value, intensives, opalescence, and characterizations [11]. However, the last and most important step to achieve a natural looking composite restoration, namely blending with the surrounding tissues, is represented by the finishing, which produces macro- and micro-morphology (superficial texture), and polishing phases. These final steps are time-consuming and require great clinical skills by the dentist, who has to closely replicate the technician’s wax-up. Using the PETG template, the proposed method allows for easily, quickly, and predictably transferring the wax-up morphology to the last labial composite layer. Upon template removal, the restoration is almost complete, as both the transition lines and surface texture are already well defined. Moreover, prolonged composite polymerization through the PETG template, in the absence of atmospheric oxygen, results in a better cured final composite layer (with lower surface roughness) that needs little to no polishing [17,18].

The proposed SST is an extremely versatile technique, covering a broad spectrum of clinical scenarios. It represents a valid alternative in all clinical cases that are considered eligible for indirect veneers (with or without tooth preparation):

- anterior teeth to be lengthened, widened, and/or re-shaped;
- anterior crown fractures;
- congenital tooth malformation;
- enamel hypocalcification;
- discolored vital teeth not responding to bleaching.

STT has no further specific contraindications beyond those already applied to indirect veneers.

Several methods have been reported in the literature to simplify and speed up finishing and polishing phases on the basis of the wax-up. Among them, the so-called “injection molding technique” or “injectable composite resin technique” is the most used [8,9]. According to this technique, a transparent silicone index is produced on the basis of the wax-up, taking care to include perforations at the incisal edge through which the tip of a flowable composite syringe is inserted and the material is injected. However, due to the inherent silicone index volume and size, this technique hinders the proper rubber dam placement (which is instead allowed by SST), thus forcing the clinician to rely only on Teflon tape and gingival retraction cords for isolation. Moreover, the injection molding technique requires the use of a flowable composite, whose reduced consistency and increased flow enhance wettability and adaptation on dental surfaces during its injection, even without excessive external pressure, as it is imposed by the low rigidity of the transparent silicone index [8]. Despite the fact that many disadvantages of the traditional flowable composites have been partially overcome with the recent introduction of the so-called “highly filled flowable resin composite” [19,20], some limitations regarding their wear resistance, polishability, and color stability still remain [9,21,22]. Problems of discoloration after 1 year of service were reported with the injection molding technique [9], while the esthetic results achieved with SST were well preserved after 1 year follow-up, without any need for new adjustments or re-polishing procedures. Finally, the injection molding technique forces the clinician to choose a single shade for the whole restoration, thus losing the undeniable esthetic advantages of the traditional layering procedure with packable composites. With SST, on the other hand, the template is used only for the last labial layer. Moreover, the inherent rigidity of the 1 mm thick PETG template (in place of the less stiff transparent silicone), paired with the lower viscosity of the warm composite, allows for gently applying the pressure needed for template seating and for excess material outflow without any distortion.

Besides the injection molding technique, another protocol for easier and more predictable direct composite veneers has been described in the literature. Created for use in the full mouth rehabilitation of worn patients, the so-called “index technique” [10] relies on the use of warm composite and transparent silicone. Similar to injection molding, this technique starts with the manufacturing of a transparent silicon index based on the diagnostic wax-up. The index is then cut for each tooth to be restored, thus obtaining individual silicone indexes to be used one at a time. After protecting the adjacent teeth with steel matrix bands and performing adhesive procedures, the warm composite is placed on the tooth to be restored. At this point, the individual index is placed on the tooth and finger pressure is applied labially and palatally to allow the outflow of excess composite through special holes [10]. Like SST, the index technique differs from injection molding in its use of a warm packable composite instead of a flowable composite. In addition to decreasing the viscosity and, therefore, enhancing the outflow [23], pre-heating the composite could enhance adaptation and minimize leakage [24,25]. Despite these advantages, however, the index technique showed remarkable inaccuracy in its ability to reproduce the diagnostic wax-up in vitro, especially in the incisal and middle-third of the anterior teeth [26].

This could be attributed to the lack of standardization in the procedure: the absence of vertical stops, in fact, could force the clinician to search for the perfect index fit by relying on finger pressure alone. SST can overcome these limitations, in which the rigidity of the PETG template ensures a repeatable, non-operator dependent fit.

Lastly, like the injection molding technique, in the index method, all of the composite material is applied at once, thus not allowing the traditional composite layering permitted by SST.

## 4. Conclusions

When dealing with anterior esthetic restorations, SST represents a valid strategy to accurately reproduce the technician’s wax-up. In the reported case, SST allowed for performing complex layering of different composite shades, under absolute isolation with a rubber dam, and with minimal need to refine and polish the restorations.

## Data Availability

The original contributions presented in the study are included in the article and Supplementary Materials, further inquiries can be directed to the corresponding author/s.

## Supplementary Materials

The following supporting information can be downloaded at: https://www.mdpi.com/article/10.3390/biomimetics9050299/s1, Video S1: Stratified Stamp Technique.
