# Supplementary material for: Anterior Esthetic Restorations with the Stratified Stamp Technique: A Case Report
Source: Biomimetics (Basel). 2024 May 18;9(5):299. doi: 10.3390/biomimetics9050299 (PMC11117549; doi:10.3390/biomimetics9050299)
Supplement: Supplementary file 1 [file biomimetics-09-00299-s001.zip › biomimetics-2951296-supplementary.pdf]

Captions to the clinical procedures shown in video as supplementary material and corresponding time references (min:sec)

00:00 – 01:00	Fractured enamel was rounded with a fine grit bur and finished with a silicone disk.
01:00 – 02:55	Teeth areas to be subjected to Selective Enamel Reduction were marked with a permanent marker, then reduced with a flame fine grit bur and polished with a silicone disk.
02:55 – 03:35	Retraction cords were placed and rubber dam isolation was performed.
3:35 – 04:30	Adhesive procedures were performed.
04:30 – 06:00	Palatal composite shells were made using a silicone index made from the diagnostic wax-up. Composite shells were then finished with a silicone disk.
06:00 – 08:45	Traditional layering procedures were performed. Two layers of dentinal core were stratified in sequence in order to progressively reduce the chroma towards the incisal margin. Care was taken to sculpt the mamelons. No freehand stratification of the final buccal layer was performed. Excess composite was removed with a silicone disk.
08:45 – 10:35	After protecting teeth 1.1 and 2.2 with teflon tape, the PETG template fit was confirmed. Adhesive was applied on tooth 2.1, then air thinned. A small amount of of enamel composite warmed up to 55° C was loaded in the acrylic PETG template on tooth 2.1. Then the template was slowly inserted and pressure was progressively applied in order to allow the full sitting and the complete outflow of any excess material prior to prolonged polymerization.
10:35 – 11:45	At the template removal, interproximal and cervical flash material was carefully removed with the use of a n. 15C surgical blade, metal, and polyester interproximal strips.
11:45 – 13:50	The same procedure was repeated for tooth 1.1, while protecting 1.2 and 2.1 with teflon.
13:50 – 16:15	Upon rubber dam removal, further interproximal and cervical finishing was performed, occlusion was checked and polishing procedures were performed just on the palatal side.
